# Textile Electrodes for EEG Recording — A Pilot Study

**DOI:** 10.3390/s121216907

**Published:** 2012-12-07

**Authors:** Johan Löfhede, Fernando Seoane, Magnus Thordstein

**Affiliations:** 1 School of Engineering, University of Borås, 501 90 Borås, Sweden; 2 School of Technology and Health, KTH-Royal Institute of Technology, 141 52 Huddinge, Sweden; E-Mail: fernando.seoane@hb.se; 3 Institute of Neuroscience and Physiology, Sahlgrenska Academy, Sahlgrenska University Hospital, 413 45 Göteborg, Sweden; E-Mail: magnus.thordstein@neuro.gu.se

**Keywords:** smart textiles, textrodes, electrodes, EEG

## Abstract

The overall aim of our research is to develop a monitoring system for neonatal intensive care units. Long-term EEG monitoring in newborns require that the electrodes don’t harm the sensitive skin of the baby, an especially relevant feature for premature babies. Our approach to EEG monitoring is based on several electrodes distributed over the head of the baby, and since the weight of the head always will be on some of them, any type of hard electrode will inevitably cause a pressure-point that can irritate the skin. Therefore, we propose the use of soft conductive textiles as EEG electrodes, primarily for neonates, but also for other kinds of unobtrusive long-term monitoring. In this paper we have tested two types of textile electrodes on five healthy adults and compared them to standard high quality electrodes. The acquired signals were compared with respect to morphology, frequency distribution, spectral coherence, correlation and power line interference sensitivity, and the signals were found to be similar in most respects. The good measurement performance exhibited by the textile electrodes indicates that they are feasible candidates for EEG recording, opening the door for long-term EEG monitoring applications.

## 1. Introduction

Cardiovascular and respiratory parameters are routinely monitored when a person is in need of intensive care. The function of the organ most vital to the eventual recovery of the patient as an individual, the brain, is however often neglected. To monitor the function of the brain itself, the most direct way is to measure the electroencephalogram (EEG), a measure of the electrical signals produced by the brain. This makes possible continuous monitoring of the brain over a long time with high time resolution. Recently developed automatic classification methods can be used to simplify the interpretation of the complex EEG signals [1–3].

**Figure 1.**
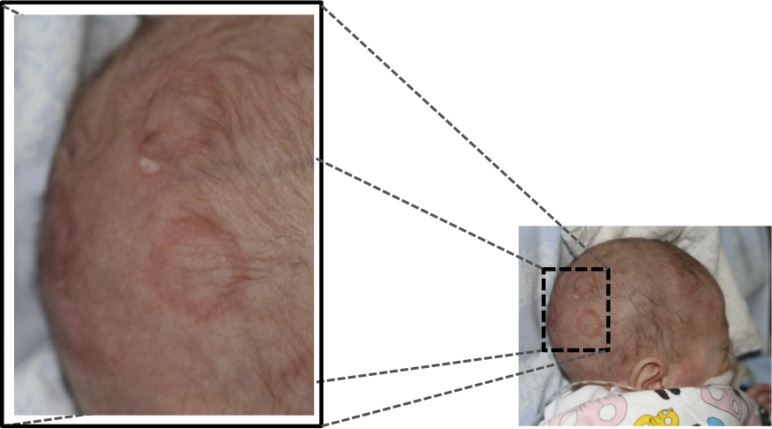
Pressure marks observed on the scalp of a neonate after wearing an electrode cap for approximately one hour.

Traditional brain monitoring systems e.g., CFM/aEEG [4] usually relies on a limited number of channels and displays a compressed and filtered version of the EEG. However, important pathological brain activity, e.g., seizures, can be highly localized and using too few electrodes may cause these events to be missed by the classification system. Having access to the underlying EEG has been shown to improve the accuracy of the classification [5]. Using traditional electrodes that are placed one by one on the scalp and attached by gel is time consuming and technically demanding work. Instead, electrode caps can be used. There is a limited selection of electrode caps for newborns, utilizing plastic cups containing the gel and the actual electrode [6]. However, the pressure of the cup onto the scalp may cause skin irritation when used during long-time monitoring (longer than one or a few hours), especially in preterm infants, see Figure 1.

To avoid the problem with pressure-points in the electrode cap we propose a solution where the cup and electrode are replaced by a patch of soft conductive textile. Textile electrodes, also known as “textrodes”, have previously been used for, e.g., ECG monitoring [7], where the signals are in the range of millivolts rather than microvolts as in the EEG case. Polymer foam covered with conductive textile [8] or a thin silver layer [9] have recently been demonstrated to work as EEG electrodes, and “water-based” electrodes made of cotton soaked in tap water have been proposed for brain-computer interface applications [10]. Having the electrodes solely made out of textile has the advantage that they can be integrated into a textile cap in a single process, making it a robust and inexpensive construction.

As a first step in the development of an electrode cap for EEG monitoring, this study aims at investigating the signal characteristics of two types of textile electrodes compared to conventional high-quality electrodes. Since our initial experiments showed that the textile electrodes did not work as dry EEG electrodes a contact medium was necessary; therefore standard electrode paste was used. Because textiles, unlike metal plates, can absorb water and stay damp for some time, physiological saline (NaCl) solution was also included as contact medium in a separate series of experiments. These tests were simple, investigating if conductive textile electrodes, applied without any skin preparation can be used to confidently record EEG signals. The aim was not to produce the best EEG signals possible, but to see if these electrodes can be used to improve the clinical situation by enabling simple and comfortable EEG caps.

## 2. Methods

### 2.1. Measurement Setup

**Figure 2.**
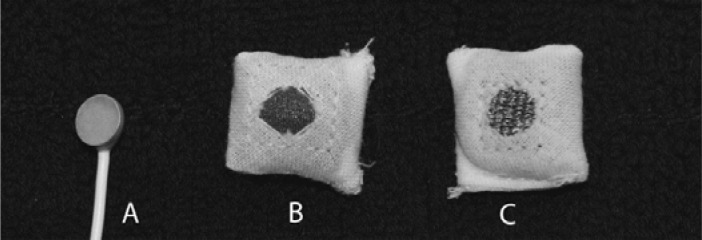
(**a**) Standard electrode. (**b**) Textile electrode Type I. (**c**) Textile electrode Type II.

**Figure 3.**
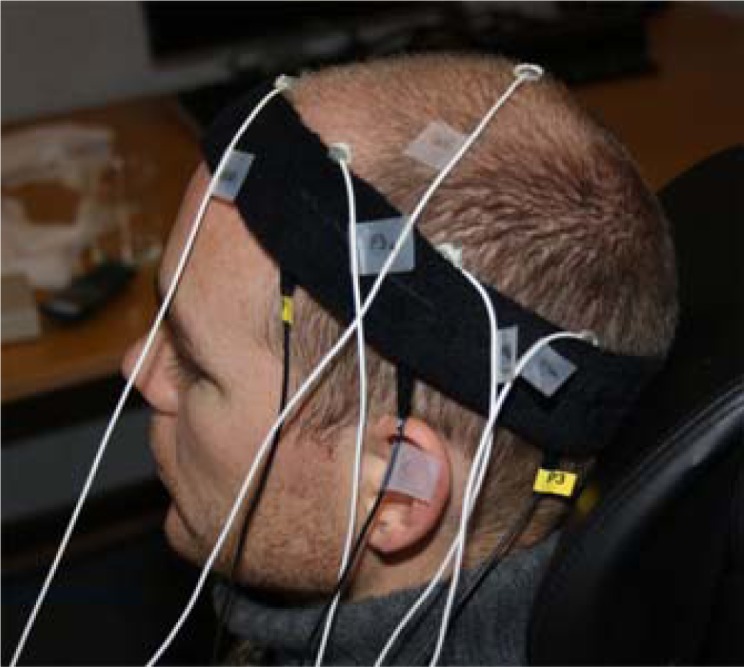
Experimental setup. The white leads are connected to standard electrodes; the black ones are connected to textile electrodes.

A tight-fitting headband was used to hold the electrodes in the approximate locations F3, C3 and P3 according to the international 10–20 system. The textile electrodes, shown in Figure 2, were tested using both physiological saline solution and standard electrode gel. The electrodes were padded in order to ensure a smooth surface and a soft pressure. They were connected using cables with crocodile clips and snap buttons. Standard high-quality sintered eight mm EEG electrodes were placed within five mm of the textile electrodes using the same standard electrode gel. Standard electrodes were also used for reference and ground and placed at the vertex and on the forehead, respectively. The setup can be seen in Figure 3.

### 2.2. EEG Recording

The signals were digitized at a sampling frequency of 1,000 Hz using a DC-coupled EEG amplifier (NeuroS4U, Medical Computer Systems, Moscow, Russia) and a laptop running the SACS^®^ signal acquisition and archiving system [11]. After recording, the signals were exported to Matlab where DC offset was removed by detrending, *i.e.*, subtracting a fitted straight line from the data. For the time domain plots and the correlation computations, power line interference was reduced using a comb filter that removed 50 Hz and its overtones and the signals were band-pass filtered from 1.5 to 70 Hz.

EEG traces were recorded from five subjectively healthy adult volunteers. The subjects were awake, sitting comfortably, with closed eyes. Under these circumstances the alpha rhythm (7.5–13 Hz) is readily identified over the posterior parts of the head. Data was recorded using standard electrodes and two different types of textrodes (*cf.* above) and each type was tested using both saline solution and electrode gel as contact medium. The recording length for each category ranged from 2.7 min to 7.4 min. Ethical approval (nr 274-11) was obtained from the regional ethical review board in Gothenburg. All test subjects gave informed consent to participate in the study.

### 2.3. Performance Comparison

The signals from the standard and textile electrodes were initially compared using visual inspection of the data in the time and frequency domains. An experienced electroencephalographer (MT) deemed the acquired signals to be of cerebral origin and of good enough quality to justify further comparisons. Thereafter, the power spectra (0–45 Hz) were estimated using the Welch method. The Welch method was applied using eight modified periodograms with a Hamming window and 50% overlap [12] and based on data epochs of 10 s. No filtering except detrending was applied on the data before spectrum estimation.

The level of similarity between the signals in the time domain using the textile and standard electrodes was estimated by calculating the Pearson product-moment correlation coefficient (corrcoef in Matlab). All data, except for parts containing very large motion artifacts, was divided into epochs of ten seconds each. The correlation was calculated for each epoch individually and then the mean of these values was calculated, thus providing a robust estimate of the actual correlation for the patient and measurement setup. Both the individual values for subjects and electrode type and the mean overall subjects and per electrode type were calculated. The RMS error was calculated in the same way as the correlation, by dividing the data into ten-second epochs.

To measure the level of similarity between the signals in the frequency domain the coherence spectra were calculated using the *mscohere* function in Matlab. This algorithm estimates the magnitude squared coherence function using Welch’s overlapped averaged periodogram method [12], and provides a measure of how well two signals correspond to each other at each frequency. The aim of the present study was to evaluate the recording capabilities of textile electrodes compared to standard ones. Therefore, the data from the subject with thick hair was excluded, because this had much lower quality than that from the other subjects.

Sensitivity to power line interference (50 Hz) was estimated by computing the power spectrum based on 120 s of data and measuring the peaks at 50 Hz, for textile electrodes and standard electrodes, respectively. Then the difference in magnitude was calculated in dB.

## 3. Electrodes

**Figure 4.**
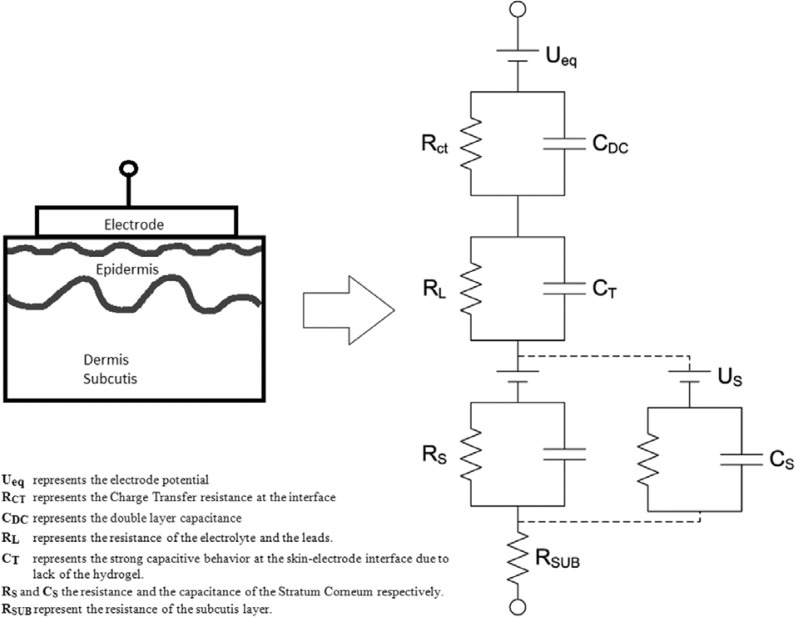
Equivalent circuit model for the skin-electrode interface.

This study is based on the assumption that the impedance of the skin-textrode interface can be represented by the circuit equivalent model proposed in [13] as shown in Figure 4 and on the previously described circuit equivalent models for the electrode-electrolyte interface [14] and the skin model [15]. When used as dry electrodes, the skin-textrode interface present a large capacitive behavior [13] modeled by C_T_. The impedance of the skin, dominated by the stratum corneum, is modeled by the parallel bridge R_S_-C_S_. C_T_ in combination with R_S_-C_S_ accounts for most of the high impedance presented by dry electrodes in contact with the skin. When wet, a resistive pathway between the textrode and the skin is created and the stratum corneum is gradually wetted through, leading to a reduction of the capacitive behavior.

Since human sweat containing around 0.1–0.4% NaCl [16] works as a weak electrolyte, sweat produced underneath the textrode should decrease the impedance of the skin-electrode interface [17–19]. The decrease in the impedance of a dry electrode is obtained in two ways [20]: The presence of sweat creates an additional conductive pathway R_L_ parallel to C_T_[13], and the availability of sweat hydrates the underlying skin reducing the value of R_S_ as reported in [21]. The same result can be obtained by wetting the textrodes with water or an electrolyte prior to their application. It has been reported that this reduces the noise and works almost as well as hydrogel [22].

### 3.1. Performance Comparison

For comparison as well as for ground and reference, standard high-quality sintered eight mm EEG electrodes (In Vivo Metric, Healdsburg, CA, USA) were used, Figure 2, with standard electrode gel (Nihon Kohden Elefix, Nihon Kohden Europe, Rosbach, Germany).

### 3.2. Textile Electrodes

**Figure 5.**
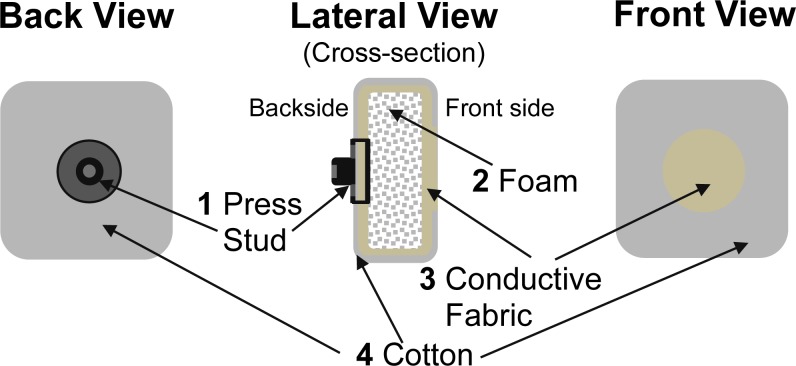
Schematic structure of the textrodes used in this study.

Since the goal of the study was to assess the feasibility of using textrodes to obtain EEG recordings, the design and the implementation of the textrode was not a priority and the structure selected was relatively simple but effective. Using a layered structure with conductive and non-conductive fabrics the electrode has been confectioned as shown in Figure 5. A layer of foam (2) has been placed between the folded layers of conductive fabric (3) to ensure that a conductive layer of fabric is pressed against the skin on the front side of the textrode. Another reason to include the foam is to avoid any mechanical influence from the press-stud (1) placed on the backside of the textrode. A non-conductive fabric, made of cotton (4), with a centered hole on the front side has been placed above the conductive fabric (3) to make the conductive textrode surface in contact with the skin of similar size as the surface of the silver electrodes used in the study. When used with gel, just enough gel to cover the cut-out in the cover fabric was utilized. When used with saline, the textrodes were soaked in the solution and then wrung out so that they were damp.

#### 3.2.1. Textile Electrode Type I

The material was knitted using a yarn containing 78% polyamide and 22% elastomer. The fabric was plated with 99% pure silver (SHIELDEX^®^ Technik-tex P130+B, Statex Produktions & Vertriebs GmbH, Bremen, Germany).

#### 3.2.2. Textile Electrode Type II

The material was made by 15% nylon, 30% silver plated conductive fibers, 20% Spandex and 35% polypropylene. The material was knitted, and is similar to terrycloth (Textronics, Inc., Wilmington, DE, USA).

## 4. Results

**Figure 6.**
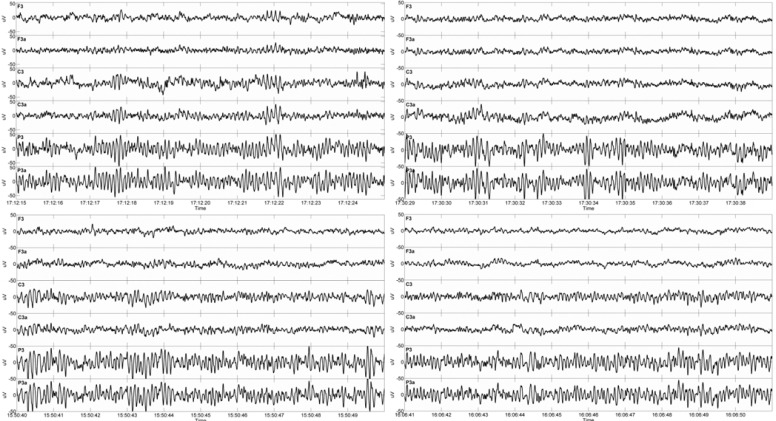
Example of EEG signals in the Matlab environment. Ten seconds of EEG with amplitude scale ±50 μV. Pair wise display of signals from the three pairs of standard (F3, C3, P3) and textile (F3a, C3a, P3a) electrodes. Top left: Type I with gel, top right: Type I with NaCl, bottom left: Type II with gel, bottom right: Type II with NaCl.

Figure 6 shows a comparison of signals in the time domain from one subject, acquired using the two different textile electrode types and with NaCl solution and gel as contact medium, respectively. With some minor exceptions, to the naked eye, the signals from the electrode pairs are remarkably similar.

**Figure 7.**
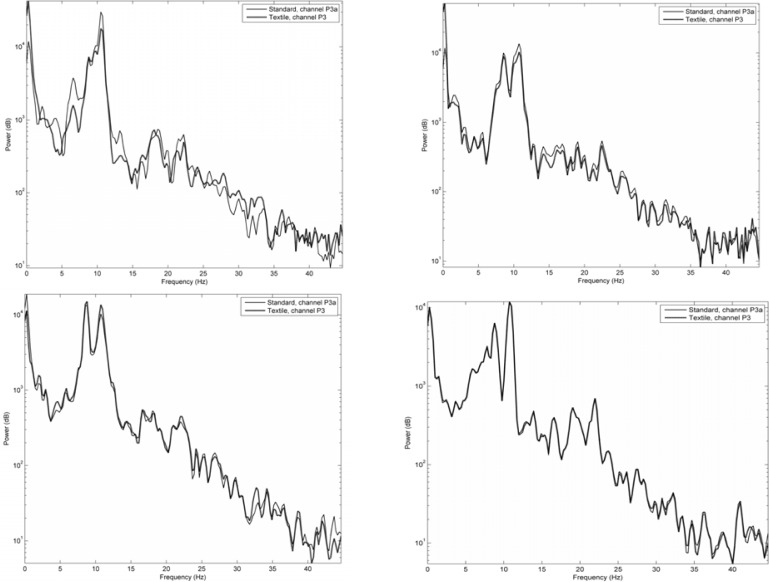
Frequency spectra for the 0–45 Hz spectrum, no filtering except detrending. Top left: Type I with gel, top right: Type I with NaCl, bottom left: Type II with gel, bottom right: Type II with NaCl.

Figure 7 shows signals acquired in the same way as in Figure 6, but here given as frequency spectra. Signals acquired using the two different textile electrode types from the P3 area using the two types of contact media are compared to those of standard electrodes. As can be seen, also the frequency spectra demonstrate a remarkable similarity between the signals.

Table 1 shows the difference in magnitude of the 50 Hz peak per electrode location type. This shows that the textile electrodes were indeed more sensitive to powerline interference when placed on hairy skin (C3 and P3). For the hairless F3 site the opposite was true; Type I with NaCl and Type II with gel were less sensitive to power line interference than the corresponding standard electrodes.

**Figure 8.**
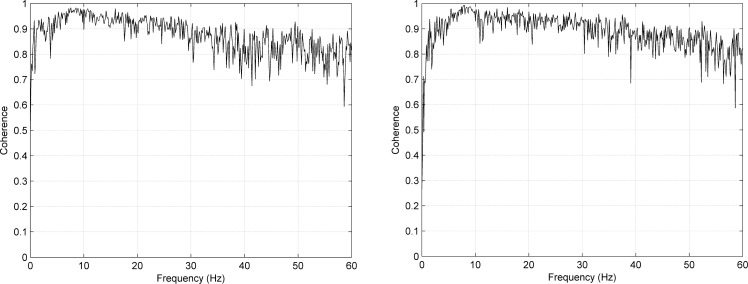
Coherence spectra for channel P3, no filtering except detrending. Left: Type I with NaCl, right: Type II with NaCl. The figures show the mean coherence over four subjects.

Figure 8 shows the mean coherence spectra for Type I and II, indicating how well the signals acquired using textile and standard electrode match for different frequencies.

Tables 2 and 3 show the correlation between textile and standard electrodes for the different test subjects and for the gel and NaCl test cases. The grand averages for the correlations were 0.82 with gel and 0.88 with NaCl for the electrode Type I and 0.82 with gel and 0.83 with NaCl for electrode Type II. The correlations were as high or higher using NaCl as when using gel as contact medium. Tables 4 and 5 show the corresponding RMS errors.

When asked to compare how comfortable the textrodes were compared to standard electrodes, the subjects did not report any significant differences.

## 5. Discussion

The main goal of our research is to improve brain monitoring in neonates under intensive care [2,3]. This requires electrodes that are easy to attach and do not harm the baby that is being monitored, even when used during several days. Existing caps may apply too much pressure on the skin of the neonate, potentially causing skin problems when used for prolonged time periods. To facilitate long-term monitoring of neonatal EEG, the use of soft textile electrodes that can be integrated in a cap that only applies a soft pressure at the electrode sites would be advantageous. There was no significant difference in the subjective appraisal of comfort comparing textrodes and standard electrodes. The reason for this is probably that the recordings were short and that the subjects were supine, *i.e.*, no pressure were applied to the electrodes apart from that of the head band used to keep them in place.

To evaluate the performance of the textile electrodes, correlations to the signals from closely placed standard electrodes were calculated. These correlations are valid since the spatial resolution of scalp EEG is rather low. This is due to the fact that a large part of the cortex underlying the electrode (about 10 cm^2^, [23]) needs to be synchronously active to make recording possible. Therefore, in practice, the neighboring electrodes had the same signal source. The correlations presented in Tables 2 and 3 show that the similarity between the signals thus acquired in most cases is high. One of the test subjects produced a much lower correlation for the C3 and P3 channels. This can be explained by the fact that this subject had longer and thicker hair than the others, making it hard to get a good connection especially for the central and parietal electrodes, while not affecting the frontal ones because of the lower amount of hair in that area. In neonates, difficulties to get high quality recordings due to the presence of large amounts of hair, is seldom a problem.

The coherence spectra presented in Figure 8 show that the mean coherence over subjects is well over 0.7 for most frequencies of interest, once again indicating that the signals acquired using textile and standard electrodes are very similar.

The issue of possibly decreased performance due to the drying of electrodes was not addressed in this pilot study only aiming at proving the principle of textile electrodes being suitable for EEG measurements. However, in a clinical setting it would be easy to regularly reapply extra saline to saline-based textile electrodes, as a part of the clinical routine in an intensive care unit. The drying could also be reduced by adding a waterproof layer on top of the conductive layer.

Our goal with this paper is to show that the weak EEG signal can be captured using soft textile electrodes, with sufficient quality for quantitative analysis. We made simple tests, investigating if conductive textile electrodes of the same size as the standard electrodes, applied without any skin preparation can be used to confidently record EEG signals. The aim was not to produce the best EEG signals possible, but to see if these electrodes can be used to improve the clinical situation by enabling simple and comfortable EEG caps. Both types of textile electrodes tested here show an encouraging performance, using both gel and NaCl as contact medium. They did not work without a contact medium, at least not in combination with the available amplifier. To decide if any of them is suitable for monitoring the long-term stability should be evaluated, for example through over-night recordings, specifically paying attention to the prevention of reduced signal quality due to the electrode drying.

The objective of the present study was to evaluate the feasibility of textrodes as a more comfortable and thereby in practice useable solution to the current alternatives in EEG-caps. The results are encouraging since to the knowledge of the authors no previous EEG recording using textrodes has been reported.

## 6. Conclusions

Our results show that soft textile materials can be used for high quality recordings of EEG signals, at least for subjects without very thick hair. We believe that this kind of electrodes could greatly improve a neonatal monitoring system. It might also be useful for e.g., ambulating monitoring of adult subjects, brain-computer interfaces or detection of drowsiness in drivers by making it more convenient and comfortable for the wearer, this way facilitating its acceptance. The presented data does not show any strong indications about which of the two textile electrode types is better. Longer recordings should be performed to determine the long term properties of the electrodes, for example if the saline will cause problems by drying out.

## Figures and Tables

**Table 1.** Textile electrode 50 Hz sensitivity (dB, mean and standard deviation; std). The two electrode types were compared to standard electrodes, using contact gel or NaCl respectively. A negative sign means that the textile electrode was less sensitive to this interference.

		**Hairless**		**Hairy**	

		**gel**	**NaCl**	**gel**	**NaCl**
**Type I**	mean	1.6	−4.9	10.0	18.8
	min	−23.9	−41.2	5.0	3.9
	max	27.4	28.0	23.6	44.3

**Type II**	mean	−11.7	2.6	1.8	15.3
	min	−37.8	−12.3	−27.1	−0.4
	max	6.8	33.6	22.4	40.1

**Table 2.** Correlation in the time domain between the three pairs of textile electrode Type I and standard electrodes using gel and NaCl, respectively (standard electrodes are denoted with an “a” after the channel name). To the right the grand average is given.

	**F3-F3a**		**C3-C3a**		**P3-P3a**		**Mean**	

	gel	NaCl	gel	NaCl	gel	NaCl	gel	NaCl
**Mean**	0.89	0.96	0.77	0.82	0.79	0.85	0.82	0.88
**Min**	0.83	0.92	0.38	0.63	0.30	0.38		
**Max**	0.96	0.99	0.96	0.92	0.98	0.99		

**Table 3.** Correlation in the time domain between the three pairs of textile electrode Type II and standard electrodes using gel and NaCl, respectively (standard electrodes are denoted with an “a” after the channel name). To the right the grand average is given.

	**F3-F3a**		**C3-C3a**		**P3-P3a**		**Mean**	

	gel	NaCl	gel	NaCl	gel	NaCl	gel	NaCl
**Mean**	0.79	0.87	0.83	0.80	0.83	0.81	0.82	0.83
**Min**	0.69	0.69	0.71	0.40	0.37	0.23		
**Max**	0.93	0.99	0.97	0.98	0.98	1.00		

**Table 4.** RMS error (microvolt) in the time domain between the three pairs of textile electrode Type I and standard electrodes using gel and NaCl, respectively (standard electrodes are denoted with an “a” after the channel name). To the right the grand average is given.

	**F3-F3a**		**C3-C3a**		**P3-P3a**		**Mean**	

	gel	NaCl	gel	NaCl	gel	NaCl	gel	NaCl
**Mean**	4.33	2.57	5.77	4.38	5.43	3.38	5.18	3.44
**Min**	3.07	0.77	2.27	3.21	1.72	0.96		
**Max**	5.49	4.26	11.8	5.59	9.24	6.69		

**Table 5.** RMS error (microvolt) in the time domain between the three pairs of textile electrode Type II and standard electrodes using gel and NaCl, respectively (standard electrodes are denoted with an “a” after the channel name). To the right the grand average is given.

	**F3-F3a**		**C3-C3a**		**P3-P3a**		**Mean**	

	gel	NaCl	gel	NaCl	gel	NaCl	gel	NaCl
**Mean**	7.55	4.16	6.23	5.52	4.19	4.72	5.99	4.80
**Min**	3.21	0.92	2.18	1.84	2.16	0.57		
**Max**	17.1	9.53	11.4	13.1	8.23	15.0		
